# A pilot study evaluating differences in muscle tissue saturation and blood flow between older adults with and without sarcopenia

**DOI:** 10.3389/fendo.2025.1644712

**Published:** 2025-08-13

**Authors:** Marni E. Shoemaker, Suzette L. Pereira, Vikkie A. Mustad, Zachary M. Gillen, Brianna D. McKay, Jose M. Lopez-Pedrosa, Ricardo Rueda, Joel T. Cramer

**Affiliations:** ^1^ College of Education and Human Sciences, South Dakota State University, Brookings, SD, United States; ^2^ Research & Development, Abbott Nutrition, Columbus, OH, United States; ^3^ Nutrition Science Consulting, LLC, Galena, OH, United States; ^4^ Department of Kinesiology, Mississippi State University, Starkville, MS, United States; ^5^ Children’s Medical Center, University of Texas Southwestern Medical Center, Dallas, TX, United States; ^6^ Abbott Nutrition Research & Development, Granada, Spain; ^7^ College of Health Professions and Sciences, University of Central Florida, Orlando, FL, United States

**Keywords:** sarcopenia, near-infrared spectroscopy, muscle blood flow, muscle tissue oxygenation, aging, exercise

## Abstract

**Introduction:**

To optimize skeletal muscle function, adequate oxygen transport and nutrient delivery to the muscle is needed. Decreased blood flow with aging may result in reduced nutritive flow to muscle, which may be compounded by those with less muscle mass. The purpose of this study was to examine differences in muscle oxygen utilization and muscle blood flow between non-sarcopenic and sarcopenic adults during pre- and post-prandial periods and during aerobic and anaerobic exercise.

**Methods:**

Ten older adults (mean±SD; age=72.4±4.9y; stature=167.5±7.6cm; body mass=71.6±12.2kg) were categorized as non-sarcopenic, and eight (age=82.9±11.4y; stature=165.7±4.5cm; body mass=70.3±8.0kg) were categorized as sarcopenic based on handgrip strength, body composition, and physical performance. Near-infrared spectroscopy (NIRS) was recorded pre-and-post consumption of a rapidly-digesting carbohydrate meal and during aerobic and anaerobic exercise. Deoxygenated hemoglobin (Hb)+myoglobin (Mb) (deoxyHb), Total Hb+Mb (THb) and muscle tissue oxygen saturation index (TSI) was measured using NIRS. Changes from baseline were calculated for deoxyHb and THb normalized to adipose tissue thickness (ΔdeoxyHb_ATT_ and ΔTHb_ATT_).

**Results:**

Post-prandial, non-sarcopenic individuals had 224% greater ΔTHb_ATT_ at 90 min (p=0.034) compared to sarcopenic and higher levels at 150 mins compared to baseline (p=0.004). Non-sarcopenia demonstrated greater ΔTHb_ATT_ at 90–120 mins than 15–60 min (p=0.018–0.047). During aerobic exercise, non-sarcopenic reported approximately 9% greater TSI compared to sarcopenic individuals (p=0.023–0.046). For anaerobic exercise, non-sarcopenic individuals saw 18%–49% lower values for ΔTHb_ATT_ at 80 and 100% compared to 60% and a 4% lower value at 100% compared to 80% of the exercise bout (p=0.034–0.043), while sarcopenic individuals experienced no change (p=0.122–0.512). Non-sarcopenia had 13% greater TSI than those with sarcopenia at 40% (p=0.026) and saw significant decreases over the anaerobic exercise bout (p=0.011–0.049) while TSI in the sarcopenic group remained unchanged (p=0.084–0.529).

**Discussion:**

Sarcopenia demonstrated decreased oxidative capacity and blood flow detectable by NIRS, potentially contributing to metabolic dysfunction. While more research is needed, NIRS responses were distinct between sarcopenic and non-sarcopenic individuals post-prandial and during exercise. Nutrition and exercise interventions focusing on strategies to improve blood flow to promote muscle health are necessary to reduce sarcopenia and related-metabolic dysfunction progression with aging.

**Clinical trials registry number:**

NCT03701867, clinicaltrials.gov.

## 1 Introduction

Aging is associated with a multitude of physiological changes including loss of skeletal muscle, in addition to changes in metabolism. Sarcopenia, defined as the age-related loss of skeletal muscle mass, strength, and function, is a multi-factorial condition that leads to an increased risk of falls, fractures, hospitalization, mortality, and decreased quality of life (1). Two potentially contributing factors to sarcopenia include poor nutrition and physical inactivity, which are linked to adequate muscle function. To optimize skeletal muscle function, there needs to be adequate oxygen transport and delivery of nutrients to the muscle. However, there is evidence showing that microvascular flow to the muscle declines with age (2), which could result in inadequate delivery of key amino acids such as leucine to the muscle, leading to decreased anabolism.

Chronic disease risk, including cardiovascular and metabolic disease, increases not only with aging, but has also been associated with sarcopenia. Reduced muscle mass has been associated with increased cardiovascular risk (3), which may cause an increased risk of macro- and microvascular dysfunction with sarcopenia, leading to inadequate blood flow and thereby inadequate nutrient delivery to skeletal muscle. Older adults have shown a 20-30% reduction in limb artery blood flow during fasted and post-prandial states compared to younger adults, theoretically related to endothelial dysfunction (2, 4). This reduction in blood flow with aging may contribute to impaired nutritive flow, thus resulting in a decrease in the transport of nutrients to the muscle, which may be compounded by those with less muscle mass, leading to the importance of examining these factors in older adults with and without sarcopenia.

Near-infrared spectroscopy (NIRS) is a technique that has recently been used to non-invasively examine vascular reactivity and muscle tissue oxygenation at rest and during exercise (5). With NIRS, relative changes in oxygenated hemoglobin (Hb) + myoglobin (Mb) (O_2_Hb) and deoxygenated Hb + Mb (deoxyHb) can be measured and used to derive total Hb + Mb (THb) and muscle tissue saturation (TSI) (%) (5). These variables have been reported to reflect oxygen delivery and extraction to provide indirect information over muscle perfusion and oxidative function (6). In particular, changes in deoxyHb has been thought to reflect mitochondrial dysfunction and detect differences in metabolic flexibility (7), while it has been hypothesized that NIRS-derived THb responses indirectly indicate changes in muscle blood flow (8). Therefore, NIRS shows promise as a non-invasive technique to examine differences in oxygenation capabilities, mitochondrial function, and vascular responsiveness, all of which may be associated with metabolic flexibility.

Additionally, skeletal muscle oxygenation responses measured via NIRS has been examined in multiple exercise and performance outcomes (9, 10). These measures are considered indirect methods to determine how well the local oxygen diffusion matches with oxygen extraction in actively contracting skeletal muscle. In general, as intensity of exercise increases, skeletal muscle oxygenation decreases in healthy populations. For example, in a ramp-incremental cycle ergometer test to VO_2_peak in young, aerobically trained and untrained healthy adults, the tissue saturation decrease was negatively correlated with VO_2_peak, while deoxyHb increase was positively correlated with VO_2_peak in the quadriceps muscles. Furthermore, THb and TSI was higher in those with higher aerobic capacity across submaximal power outputs, indicating normal responses across fitness levels (11). However, in individuals with disease states, NIRS variables may respond differently. In those with diabetes mellitus, TSI was lower at baseline and decreased more during isometric contractions in comparison to healthy individuals (9). Individuals with diabetes typically have reduced capillary density, decreased muscle blood flow at rest and during exercise, and impaired glucose metabolism, which may be contributing factors to the inability to meet oxygen demands of exercising muscle (12, 13). Similarly, muscle reoxygenation time in the recovery phase after maximal exercise was greater in older adults compared to middle-aged individuals in both sedentary and active individuals, indicating that reperfusion was slower with age regardless of training status. However, training status may alleviate some of the reduced oxygenation with age (10), possibly due to the well-established effects of exercise on increasing muscle capillarization in both young and aged muscle (14).This suggests that although reduced oxygenation and reperfusion may be normal responses to aging, promoting skeletal muscle health as individuals age could be impactful in reducing these outcomes.

Although previous studies have compared macrovascular and microvascular blood flow between young and older adults and shown significant decrements with age, there are no studies comparing blood flow and oxygen delivery between sarcopenic versus non-sarcopenic older adults. It is postulated that those with sarcopenia have greater perturbances in these normal physiological processes that are essential for muscle functionality than those without sarcopenia. Therefore, the purpose of this study was to examine if there were differences in muscle oxygen utilization and muscle blood flow between non-sarcopenic and sarcopenic adults during pre- and post-prandial periods and during aerobic and anaerobic exercise.

## 2 Materials and methods

### 2.1 Study design

**Figure 1:**
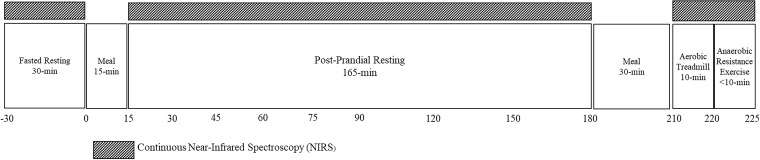
Test visit activity timeline. Near-infrared spectroscopy (NIRS) was measured for 30 minutes prior to and 180 minutes after consuming a RDC meal. After consumption of another CHO-rich meal, NIRS was measured during steady-state aerobic exercise for 10 mins and a fatiguing bout of anaerobic exercise.

Detailed information on this study protocol was reported previously (15) and is registered on clicinaltrials.gov (NCT03701867). This pilot study utilized a single center, cross-sectional design. Potentially eligible participants came to the laboratory for the Screening Visit for assessment of anthropometrics, body composition, and leg extension strength. If participants were eligible for the Test Visit, a return visit was scheduled four to seven days after the Screening Visit. During the Test Visit, muscle tissue oxygenation was monitored continuously by NIRS at rest (fasted and post-prandial response to a rapidly-digesting carbohydrate (CHO) meal), and during aerobic and anaerobic exercise. **Figure 1** displays the timeline of the Test Visit.

### 2.2 Participants

Participants were recruited from previous study databases, local community organizations, and retirement or older adult living communities. Three hundred fifty-six individuals were pre-screened for eligibility. A total of 22 participants were eligible based on previously reported inclusion and exclusion criteria and enrolled into the study (15). Participants were included in the study if they met the following inclusion criteria during the pre-screening and Screening Visit: 1) Participant is 65 years of age or older at the time of screening; 2) Participant’s body mass index (BMI) is ≥ 18.0 and ≤ 39.0 kg·m^-2^; 3) Participant is ambulatory (able to walk without assistance); 4) Participant is not a current smoker (within past 10 years); 5) Participant is classified as low OR moderate risk as defined by the American College of Sports Medicine (ACSM) Guidelines for Exercise Testing & Prescription based on the responses from AHA/ACSM Health/Fitness Facility Preparticipation Screening Questionnaire (16); 6) Participant has normal muscle mass and strength/performance (normal grip strength [≥ 30.0 kg (men); ≥ 20.0 kg (women)]) OR low muscle mass and strength/performance (low grip strength [< 30 kg (men); < 20 kg (women)]) (1); 7) If Participant is on thyroid medication or hormone replacement therapy, states he/she has been on a constant dosage for at least 2 months prior to Screening Visit; 8) Participant states he/she is willing to follow protocol as described; 9) Participant has voluntarily signed the Informed Consent Form.

Participants were excluded from the study if they met any of the following criteria during pre-screening, Screening Visit, or Test Visit: 1) Participant states he/she has a history of metabolic/endocrine (diabetes), hepatic, or renal disease, myocardial infarction, peripheral vascular disease, respiratory or neuromuscular disease; 2) Participant states he/she regularly participates in a resistance exercise program; 3) Participant states he/she has had poor appetite with recent unexplained weight loss (e.g., 10 pounds [4.5 kilograms]) over the past 6 months; 4) Participant states he/she has a current infection (requiring medication or which might be expected to require hospitalization), has had inpatient surgery, or corticosteroid treatment (excluding topical creams) in the last 3 months or antibiotics in the last 3 weeks prior to the Screening Visit; 5) Participant states that he/she has an active malignancy, excluding carcinoma *in-situ* of the cervix, cutaneous malignancies (basal cell carcinoma, squamous cell carcinoma, except melanoma); 6) Participant states that he/she has a chronic, contagious, infectious disease, such as active tuberculosis, Hepatitis A, B, or C, or HIV; 7) Participant reports currently taking medications/dietary supplements or substances that could profoundly modulate metabolism in the opinion of the principal investigator (PI) or study physician, e.g. progestational agents, steroids, growth hormone, dronabinol, marijuana, CaHMB, free amino acid supplements, dietary supplements to aid weight loss or gain. Exceptions included use of multi-vitamin/mineral supplement, topical or optical steroids and short-term use (less than two weeks) of dexamethasone; 8) Participant is known to be allergic or intolerant to any foods; 9) Participant states he/she has had history of gastrointestinal disease (e.g., Crohn’s, colitis, celiac), or surgeries (including gastric balloon), gastroparesis, or taking medications that are known in the opinion of the PI or study physician (e.g., cholinergic agonists, prokinetic agents, opioid antagonists, antidiarrheals, and antibiotics) to interfere with consumption/digestion/absorption of nutrients; 10) Participant states he/she has an eating disorder, severe dementia or delirium, history of significant neurological or psychiatric disorder, alcoholism, substance abuse, or other conditions that may interfere with compliance with study protocol procedures in the opinion of the PI or study physician; 11) Participant states that he/she is a participant in a concomitant trial or trial of a non-registered drug (or is within the 30 day follow-up period for such a trial).

Prior to participation, all individuals reviewed, signed, and dated an Informed Consent Form approved by the university Institutional Review Board (IRB) for the protection of human subjects (IRB Project # 18543; IRB Approval Date: September 6, 2018). A signed and dated copy was provided to the participant prior to any study participation.

### 2.3 Screening visit

Classification of eligibility and sarcopenic status were determined through assessments of anthropometrics, handgrip strength, Short Physical Performance Battery (SPPB), and body composition with dual-energy X-ray absorptiometry (DXA) according to the European Working Group on Sarcopenia in Older People (1). Ultrasonography determined adipose tissue thickness of the vastus lateralis. An estimation of VO_2_max and maximal leg extension strength was determined.

#### 2.3.1 Muscle strength and function

A handheld dynamometer (Jamar^®^ Hydraulic Hand Dynamometer, Patterson Medical, Warrenville, IL) (17) was used to measure handgrip strength. Participants adducted the right arm to their side with 90°flexion at the elbow. Three trials were performed, with the average of the trials used as the final strength value. The SPPB evaluated balance, gait speed at four meters, and lower body endurance with a chair stand test based on the protocol developed by Guralnik and colleagues (18). The balance component tested the participant’s balance at three progressively harder values. A score was provided based on completion or time to failure. For gait speed assessment, participants were instructed to walk four meters at their normal walking speed. The test was timed twice. If the second gait speed trial was more than 10% different than the first trial, a third trial was conducted. For the chair stand, participants were instructed to start seated on a chair with their feet firmly planted on the ground and their arms crossed on their chest. Participants were then timed to see how long it took to rise from the chair and return to the seated position five times. Each test was scored based on level of completion of assigned task, and a total score of all three tests is calculated. The range of score is from 0 – 12, where 0 is the lowest and 12 is the highest level of functionality.

#### 2.3.2 Anthropometrics and body composition

Height and body mass were measured using calibrated digital scale and stadiometer (Seca 769, Hamburg, Germany) to calculate body mass index (BMI). Waist circumference was measured. Whole-body DXA (Lunar iDXA, GE Healthcare, Madison, WI) determined fat mass (FM, kg), fat-free mass (FFM, kg), percent body fat (BF%), and appendicular lean soft tissue (ALST). Participants’ bodies were free from any external metal objects, and any internal metal devices were noted. Participants were instructed to lie supine on the padded scanner table with their hands pronated and near their body, but without touching the hip. The appropriate thickness (thin, standard, or thick) based on chest depth was selected prior to completing each total-body scan (Lunar iDXA User Manual, GE Healthcare, Madison, WI). Participants were categorized as “normal” or “low” skeletal muscle mass based on relative skeletal muscle index percent (RSMI%) from Kim et al. (19) and Janssen et al. (20).

#### 2.3.3 B-mode ultrasound

Transverse images were taken at the site of the NIRS device at 66% of the distance from the anterior superior iliac spine (ASIS) to the superior border of the patella (21). Ultrasound images were taken using a portable brightness mode (B-mode) ultrasound-imaging device (GE Logiqe, USA) and a multi-frequency linear-array probe (12L-RS; 5–13 MHz; 38.4 mm field-of-view) (21). Participants were positioned on a plinth in the supine position with their legs extended and supported on the plinth with feet braced. Water-soluble transmission gel was applied to the skin to enhance acoustic coupling and reduce near field artifacts. Equipment settings were optimized for image quality with a gain of 58 dB and a frequency of 12 MHz. These settings were held constant across participants. Image depth was adjusted based on each participant’s leg size and was then held constant for each participant. Images were taken until three images of acceptable quality were acquired. Images with the highest visual contrast were used for analysis. Images were analyzed using Image-J Software (National Institutes of Health, USA, version 1.52v). Prior to analysis, images were scaled from pixels to cm using the Image-J straight-line function. Subcutaneous fat (cm) over the NIRS placement site was quantified using the straight-line function that included as much of the subcutaneous fat as possible while excluding the VL (21).

#### 2.3.4 O_2_max estimation

A non-exercise estimation O_2_max was performed using the following regression equation (**Equation 1**) (22):


(1) $$\begin{array}{c} \dot{V}O2max\;=\;59.416\;-\;\left(0.327\;x\;age\;\left(y\right)\right)\;+\;(11.488\;x\;\\ sex)\;+\;\left(1.297\;x\;PASS\right)\;-\;\left(0.266\;x\;waist\;girth\;\left(cm\right)\right) \end{array}$$

Where the physical activity status scale (PASS) represents a score ranging from 0 to 10 determined from the NASA Physical Activity Status Scale (23). Sex was dichotomized as male = 1 and female = 0. This estimation was used to calculate 50 – 60% of VO_2_max for the steady-state aerobic exercise bout.

#### 2.3.5 Leg extension strength

Maximal leg extension strength was estimated sub-maximally with a unilateral, dynamic constant external resistance five repetition maximum (5-RM) test (24). Leg extensions were completed on a plate-loaded leg extension machine (Hammer Strength Plate-Loaded, Iso-Lateral Leg Extension Machine; LifeFitness, Rosemont, IL, USA) that was custom fitted with a load cell (Omegadyne, model LCHD-500, 0-500 lb; Stamford, CT, USA). Participants were seated on a Biodex chair (Biodex Medical Systems, Inc., Shirley, NY, USA) and secured with restraining straps over the pelvis, trunk, and contralateral thigh. Participants were instructed to sit upright with their back against the chair and to tightly hold the handles located near their hips. The lateral epicondyle of the right femur was aligned with the axis of rotation of the leg extension device.

Participants completed a warm up set of 10 repetitions with 4.5 kg external resistance. After a two-min rest period, an appropriate amount of weight was added for a first attempt to find a 5-RM. Two to five minutes rest was allowed between attempts. The set was determined as a successful 5-RM if the participant was able to complete the five repetitions through their full range of motion, but not able to complete a sixth repetition. Once the 5-RM was determined, the external resistance added was recorded and used to estimate the 1-RM using the following equation (r=0.994) (**Equation 2**) (24):


(2) $$\begin{array}{c} 1-RM\;=\;1.0970\;x\;\left(5-RM\;loaded\;resistance\;\left(kg\right)\right)\;\\ +\;14.2546 \end{array}$$

### 2.4 Test visit

Participants who qualified for the Test Visit were scheduled to arrive at the laboratory four to ten days after the Screening Visit after an 8 – 16 hour fast.

#### 2.4.1 Near-infrared spectroscopy

For assessment of muscle tissue oxygenation during rest and exercise (25), a portable, continuous wavelength NIRS device (PortaMon MKII, Artinis, Einsteinweg 17, Netherlands) was positioned over the right vastus lateralis muscle at 66% of the distance from the ASIS to the superior border of the patella. Prior to placement, the local skin area was prepared by light dry shaving, light abrasion with a cotton towel, and cleaned with isopropyl alcohol. The NIRS device was covered in a thin, transparent plastic wrap as protection from any fluids. The device was placed on the muscle with a black cloth covering it and then secured to the skin with dark-colored, opaque self-adherent Coban™ wrap (3M™, Maplewood, MN, USA), in order to limit the effects of peripheral light disrupting the sensor.

The NIRS device measures relative concentration changes from baseline in oxygenated hemoglobin (Hb) + myoglobin (Mb) (O_2_Hb) and deoxygenated Hb + Mb (deoxyHb). Muscle tissue oxygen saturation index (TSI) (%) was measured using spatially-resolved spectroscopy to obtain absolute values. From the modified lambert-Beer law, relative concentrations of O_2_Hb and deoxyHb can be obtained and used to calculate THb, where THb = O_2_Hb + deoxyHb (26). Three light emitting diodes each transmit two wavelengths (760 and 850 nm) of light through the skin, which scatters back and is received by the receiver. The transmitting diodes and receiver are designed spatially to provide three source-detector distances of 30 mm, 35 mm, and 40 mm, respectively, to allow for a depth sensitivity of ~1.5 cm (27). The NIRS device recorded TSI, O_2_Hb, deoxyHb, and THb 30 min prior to and 180 min post consumption of a standard rapidly-digesting CHO (RDC) meal which included half of an English muffin, 1 Tbsp peanut butter, and Gatorade^®^, with a final composition of 51 g carbohydrates, 9 g fat, and 6 g protein (304 calories) (**Figure 1**). Measurements were averaged over 15-min windows within the 15 min timepoints and over the last 15 min of the 30 min timepoints over the resting period. All NIRS measurements were normalized for adipose tissue thickness (ATT) as measured by ultrasonography at the site of NIRS device placement (28). Due to interruption in the signal at multiple timepoints, four participants were excluded from this analysis (non-sarcopenic, n=1; sarcopenic, n=3).

#### 2.4.2 Submaximal aerobic exercise

A second rapidly-digesting CHO meal of 50 g CHO, 9 g fat, and 6 g protein (1 slice white bread, 1 oz cheese, and Gatorade^®^) (316 calories) was provided prior to the submaximal aerobic exercise protocol (29). The aerobic exercise protocol consisted of completing a 10-min walk (0% grade) at a steady-state, between 50% - 60% of their estimated VO_2_ max. NIRS variables were measured continuously throughout the aerobic exercise. Measurements were exported and averaged over every 2-min period.

#### 2.4.3 Anaerobic fatiguing exercise

Participants were secured with straps to the Biodex (Biodex Medical Systems, Inc., Shirley, NY, USA) chair in preparation for the submaximal anaerobic fatiguing exercise protocol. Approximately 30% of the estimated 1-RM from the Screening Visit was loaded onto the leg extension device (Hammer Strength Plate-Loaded, Iso-Lateral Leg Extension Machine; LifeFitness, Rosemont, IL, USA). Participants were instructed to complete one repetition every three seconds, with a metronome set at a tempo of 20 beats per minute for an auditory cue as to when to complete a leg extension repetition. Leg extension repetitions were completed at each metronome sound until the participant was unable to complete another repetition throughout their full range of motion. Number of repetitions and time to exhaustion were recorded. NIRS was measured continuously throughout the anaerobic exercise protocol. NIRS variables were exported and divided into quintiles (%) based on the number of repetitions and time to exhaustion and categorized as 0 – 20%, 20 – 40%, 40 – 60%, 60 – 80%, and 80 – 100% time to exhaustion (TTE).

### 2.5 Statistical analyses

Means, standard deviations (SD), and 95% confidence intervals (CI) were calculated. Changes from baseline were calculated for deoxyHb and THb normalized to adipose tissue thickness (ΔdeoxyHb_ATT_ and ΔTHb_ATT_, respectively) at rest and during aerobic and anaerobic exercise. Two-way analyses of variance (ANOVAs) (sex x sarcopenic status) compared baseline demographics, anthropometrics, body composition, strength, diet composition, and repetitions to failure. Separate two-way mixed factorial ANOVAs (time x sarcopenic status) analyzed TSI, ΔdeoxyHb_ATT,_ and ΔTHb_ATT_. Planned comparisons between non-sarcopenic and sarcopenic groups and time series patterns were determined *a priori* and performed with independent and paired samples t-tests. All statistical analyses were performed with IBM SPSS v. 25 (Chicago, IL, USA). An alpha of *p* ≤ 0.05 was considered statistically significant for all comparisons.

## 3 Results

### 3.1 Sarcopenia classification and demographics

Ten older adults (mean ± SD; age=72.4 ± 4.9 y; stature=167.5 ± 7.6 cm; body mass=71.6 ± 12.21 kg) were categorized as non-sarcopenic, and eight older adults (age=82.9 ± 11.4 y; stature=165.7 ± 4.5 cm; body mass=70.3 ± 8.0 kg) were categorized as sarcopenic. Baseline differences between sarcopenic status and sex in demographics, body composition, and muscle strength are reported in **Table 1**. There were significant main effects (sarcopenia status x sex) for age, weight, BMI, FFM, and leg extension strength (p=0.015 – 0.040). There were significant main effects for sex for height, BF%, FFM, RSMI, handgrip strength, SPPB, estimated VO_2_max, leg extension strength, and repetitions to exhaustion (p<0.001 – 0.018), in which males were taller, had less body fat, greater FFM, FSMI, handgrip strength, SPPB score, estimated VO_2_max, leg extension strength, and repetitions to exhaustion. Those with sarcopenia had lower RSMI, handgrip strength, estimated VO_2_max, leg extension strength, and repetitions to exhaustion compared to those without sarcopenia (p<0.001 – 0.044) (**Table 1**).

**Table 1:** Means ± standard deviations (SD) for baseline demographics, anthropometrics, body composition, strength, and repetitions to failure for the non-sarcopenic (n=10) and sarcopenic (n=8) older adults.

		Non-sarcopenic						Sarcopenic						Interaction effect (Sarcopenia x Sex)	Main effect (Sex)	Main effect (Sarcopenia)
		Males			Females			Males			Females					
Age	(y)	71.8	±	5.7	73.0	±	4.6	87.0	±	9.4	70.5	±	7.8	**p = 0.032**	p = 0.058	p = 0.109
Height	(cm)	172.2	±	6.0	162.8	±	6.3	167.1	±	4.3	161.7	±	2.7	p = 0.477	**p = 0.018**	p = 0.218
Weight	(kg)	80.2	±	9.5	63.0	±	7.7	69.0	±	8.91	74.3	±	3.0	**p = 0.022**	p = 0.193	p = 0.997
Body Mass Index (BMI)	(kg·m^-2^)	27.0	±	1.4	23.9	±	2.3	24.7	±	3.1	28.4	±	0.2	**p = 0.015**	p = 0.833	p = 0.364
Percent Body Fat	(%)	22.6	±	3.5	36.3	±	3.2	26.8	±	6.5	41.2	±	0.3	p = 0.882	**p < 0.001**	p = 0.078
Fat-free Mass (FFM)	(kg)	58.4	±	6.9	37.9	±	5.5	46.6	±	3.7	40.2	±	2.8	**p = 0.022**	**p < 0.001**	p = 0.100
Relative Skeletal Muscle Index (RSMI)	(%)	40.1	±	2.2	29.4	±	1.0	34.7	±	2.4	26.5	±	0.5	p = 0.215	**p < 0.001**	**p < 0.001**
Handgrip Strength	(kg)	42.1	±	8.5	25.1	±	5.2	34.7	±	2.4	25.9	±	0.7	p = 0.337	**p < 0.001**	**p = 0.001**
Short Physical Performance Battery (SPPB)	(score)	12.0	±	0.0	11.5	±	0.8	10.5	±	1.0	10.2	±	1.6	p = 0.826	**p = 0.006**	p = 0.385
Estimated VO_2_max	(ml·kg^-1^·min^-1^)	32.1	±	4.9	22.6	±	3.0	26.6	±	4.2	12.5	±	3.9	p = 0.301	**p < 0.001**	**p = 0.002**
Leg Extension Strength (5RM)	(kg)	19.5	±	1.3	9.5	±	2.8	11.7	±	3.3	7.8	±	1.6	**p = 0.040**	**p < 0.001**	**p = 0.004**
Repetitions to Exhaustion @ 30% 1RM	(reps)	26.6	±	10.1	12.8	±	3.1	14.5	±	3.6	11.0	±	1.4	p = 0.123	**p = 0.015**	**p = 0.044**
Time to Exhaustion	(mins)	1.8	±	1.0	1.3	±	0.4	1.2	±	0.3	0.8	±	0.2	p = 0.885	p = 0.126	p = 0.109

P-values are type I errors of the interactions and main effects.

Bolded p-values indicate significantly different at alpha > 0.05.

### 3.2 Postprandial muscle tissue oxygenation

**Figure 2:**
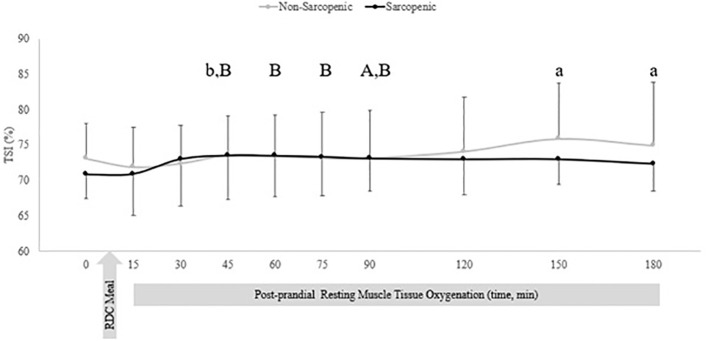
Means ± 95% confidence intervals (CI) of resting TSI (%) in non-sarcopenic (gray lines) and sarcopenic (black lines) individuals 30 min prior and 150 min post RDC meal. Between group differences is represented by an * symbol. Non-sarcopenic group within group differences is represented by lowercase letters. Sarcopenic group within group differences is represented by uppercase letters. (a) significantly higher TSI than at 15 min. (b) significantly higher TSI at 45 min compared to 30 min. (A) significantly higher TSI at 90 min compared to baseline (0). (B) significantly higher TSI compared to 15 min, (*p* ≤ 0.05).

There were no significant time x sarcopenic status interactions, but were significant main effects for time for TSI, ΔdeoxyHb_ATT_, and ΔTHb_ATT_. There were no differences between groups for TSI. For non-sarcopenic individuals, there was an increase in TSI over time, where measures at 150 – 180 min were greater than 15 min post RDC meal, and 45 min was greater than 30 min (p=0.007 – 0.029). For sarcopenic individuals, 90 mins was greater than baseline (p=0.027) and 45, 60, 75, and 90 mins were greater than at 15 mins (p=0.021 – 0.048) (**Figure 2**).

**Figure 3:**
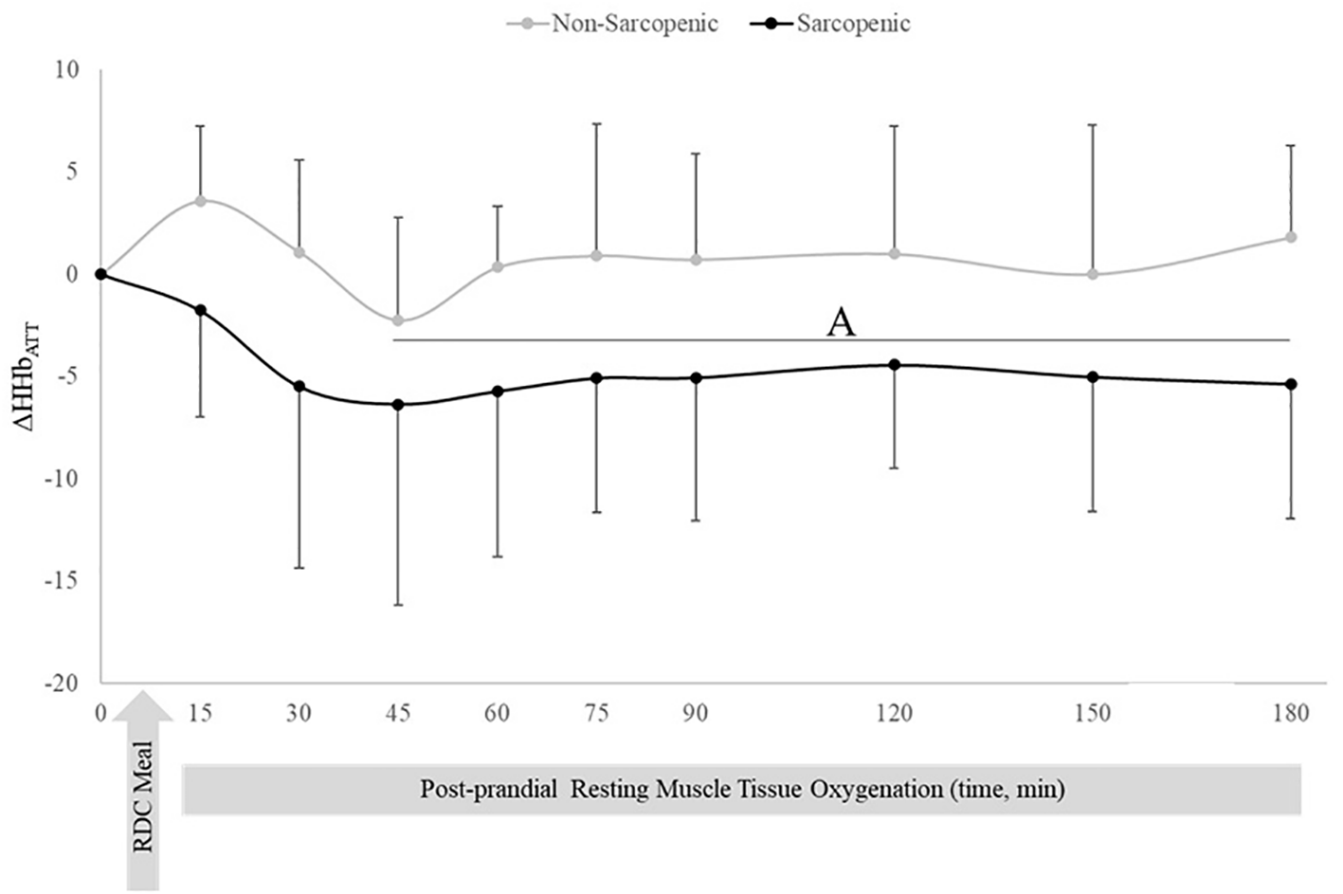
Means ± 95% confidence intervals (CI) of resting change from baseline in deoxygenated hemoglobin relative to adipose tissue thickness (ΔHHb_ATT_) in non-sarcopenic (gray lines) and sarcopenic (black lines) individuals 30 min prior and 180 min post a CHO-rich meal. Between group differences is represented by an * symbol. Non-sarcopenic group within group differences is represented by lowercase letters. Sarcopenic group within group differences is represented by uppercase letters. **(A)** significantly lower ΔHHb_ATT_ values compared to 15 min in sarcopenic individuals (*p* ≤ 0.05).

**Figure 4:**
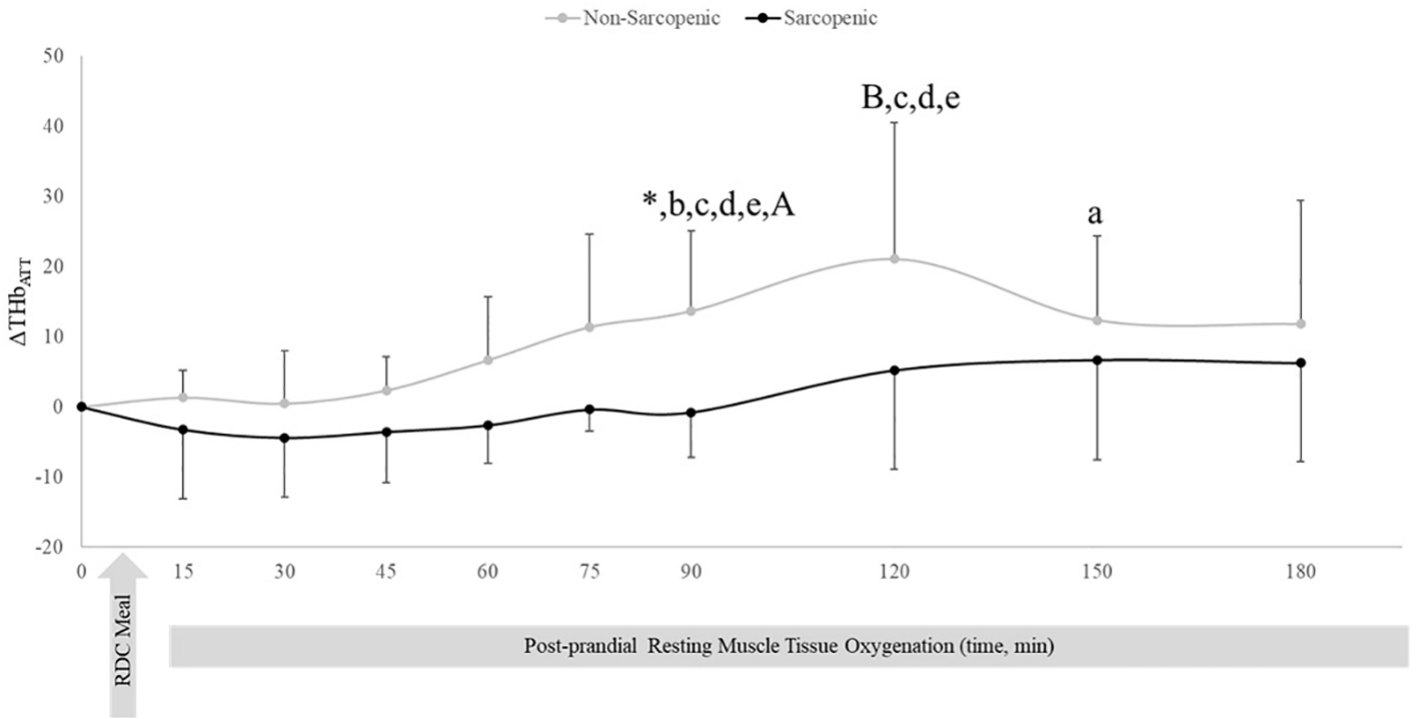
Means ± 95% confidence intervals (CI) of resting change from baseline total hemoglobin adjusted for adipose tissue thickness (ΔTHb_ATT_) in non-sarcopenic (gray lines) and sarcopenic (black lines) individuals 30 min prior and 180 min post a CHO-rich meal. Between group differences is represented by an * symbol. Non-sarcopenic group within group differences is represented by lowercase letters. Sarcopenic group within group differences is represented by uppercase letters. (*) significant difference between non-sarcopenic and sarcopenic groups at 90 min. (a) significantly higher levels than baseline. (b) significantly higher than 15 min, (c) significantly higher than 30 min. (d) significantly higher than 45 min. (e) significantly higher than 60 min. (A) significantly higher than 30 min (*p* ≤ 0.05).

There were no changes over time in ΔHHb_ATT_ for non-sarcopenic individuals; however, ΔHHb_ATT_ for the sarcopenic group significantly decreased from baseline at 45, 60, 75, 90, 120, 150, and 180 mins (p=0.003 – 0.026) (**Figure 3**). Non-sarcopenic individuals had a 224% greater ΔTHb_ATT_ at 90 min (p=0.034) compared to sarcopenic older adults and showed significantly higher levels than baseline at 150 mins (p=0.004). Non-sarcopenic participants also had greater ΔTHb_ATT_ at 90 – 120 mins than 15 – 60 min (p=0.018 – 0.047) (**Figure 4**). Sarcopenic subjects had a greater ΔTHb_ATT_ at 90 min compared to 30 mins (p=0.031) (**Figure 4**).

### 3.3 Submaximal aerobic exercise muscle tissue oxygenation

**Figure 5:**
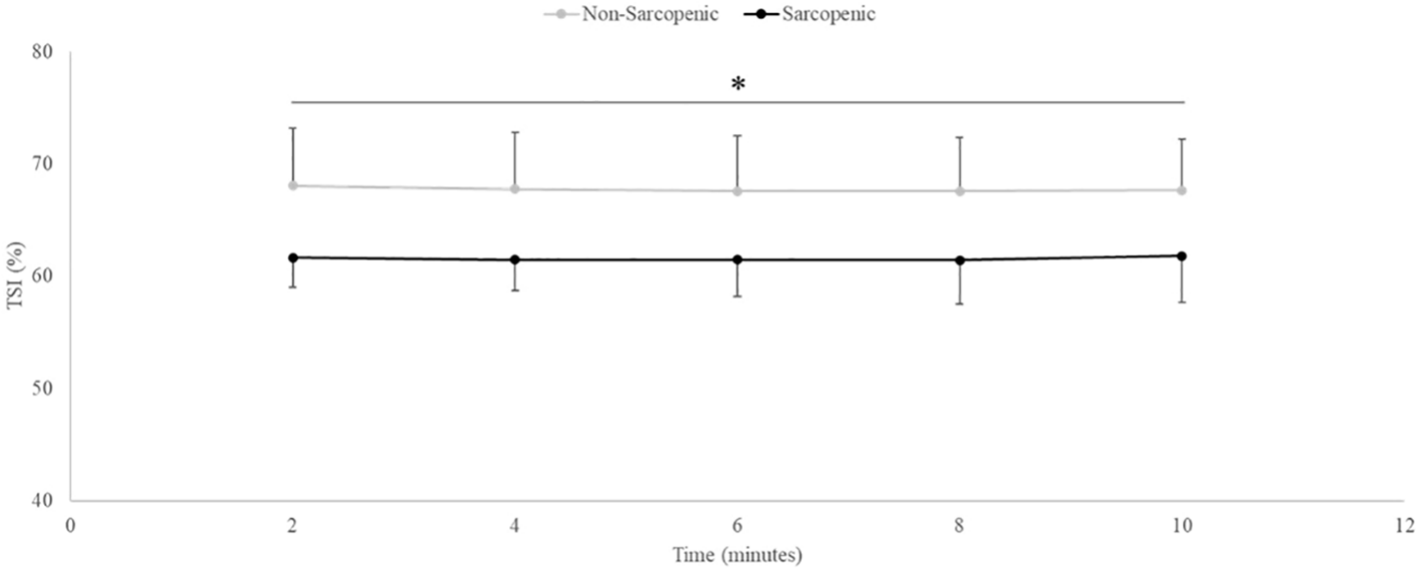
Means ± 95% confidence intervals (CI) of Tissue Saturation Index (TSI, %) in non-sarcopenic (gray lines) and sarcopenic (black lines) individuals throughout steady-state aerobic exercise. Between group differences is represented by an * symbol. Non-sarcopenic group within group differences is represented by lowercase letters. Sarcopenic group within group differences is represented by uppercase letters. (*) indicates significant difference between groups at all timepoints (*p* ≤ 0.05).

There were no significant time x sarcopenic status interactions or main effects for time. There was a significant main effect for sarcopenic status for TSI (p=0.028). Non-sarcopenic individuals had approximately 9% greater TSI throughout the entire aerobic exercise test compared to sarcopenic individuals (p=0.023 – 0.046) (**Figure 5**).

### 3.4 Anaerobic fatiguing exercise

**Figure 6:**
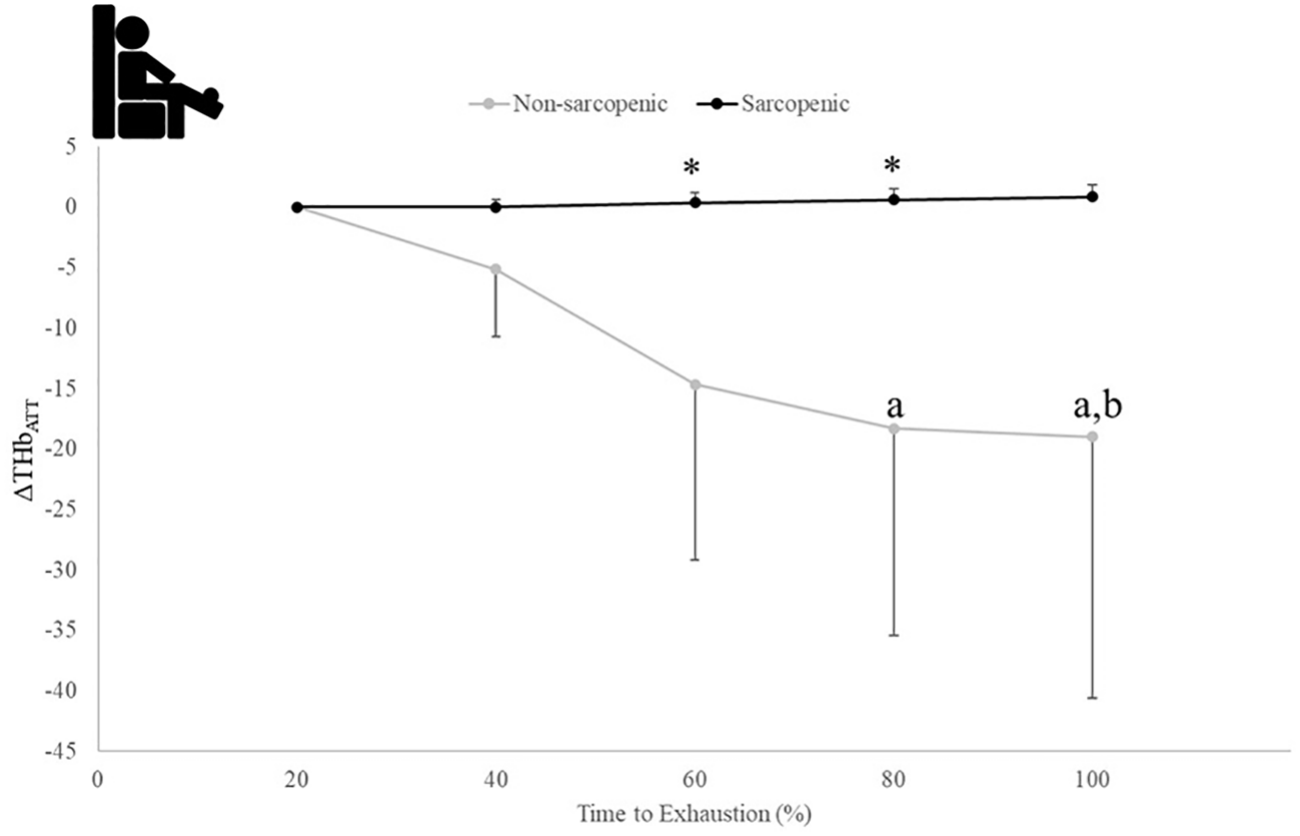
Means ± 95% CI of changes from baseline for THB (ΔTHb_ATT_) over time to exhaustion (TTE) (%) during a fatiguing bout of leg extensions in non-sarcopenic and sarcopenic older adults. Between group differences is represented by an * symbol. Non-sarcopenic group within group differences is represented by lowercase letters. Sarcopenic group within group differences is represented by uppercase letters. (*) indicates differences between groups. (a) indicates significantly less than 60%, (b) indicates less than 80% (*p* ≤ 0.05).

**Figure 7:**
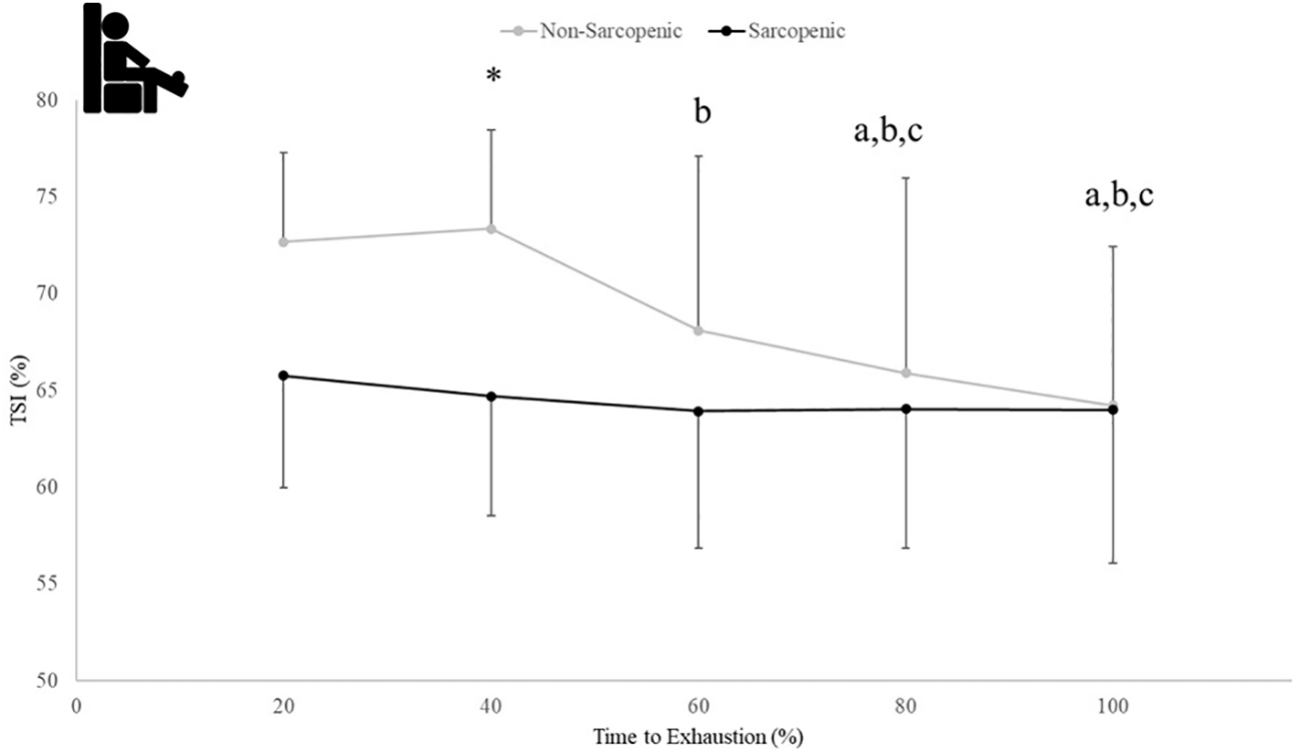
Means ± 95% CI of muscle tissue saturation (TSI) (%) over time to exhaustion (TTE) (%) during a fatiguing bout of leg extensions in non-sarcopenic and sarcopenic older adults. Between group differences is represented by an * symbol. Non-sarcopenic group within group differences is represented by lowercase letters. Sarcopenic group within group differences is represented by uppercase letters. (*) differences between groups at 40% TTE. (a) indicates less than 20% TTE, (b) indicates less than 40% TTE, and (c) indicates less than 60% TTE (*p ≤* 0.05).

There were no significant time x sarcopenic status interactions (p=0.068-0.164) but were main effects for time for ΔHHb_ATT_ (p=0.043) and TSI (p=0.021), and a main effect for sarcopenic status for ΔTHb_ATT_ (p=0.020). Non-sarcopenic individuals saw 18% - 49% lower values for ΔTHb_ATT_ at 80 and 100% compared to 60% and a 4% lower value at 100% compared to 80% of the exercise bout (p=0.034 – 0.043), while sarcopenic individuals experienced no change (p=0.122 – 0.512) (**Figure 6**). The non-sarcopenic group had 13% greater TSI than those with sarcopenia at 40% (p=0.026) and saw significant decreases over the fatiguing exercise bout (p=0.011 – 0.049) while TSI in the sarcopenic group remained unchanged (p=0.084 – 0.529) (**Figure 7**). 

## 4 Discussion

**Figure 8:**
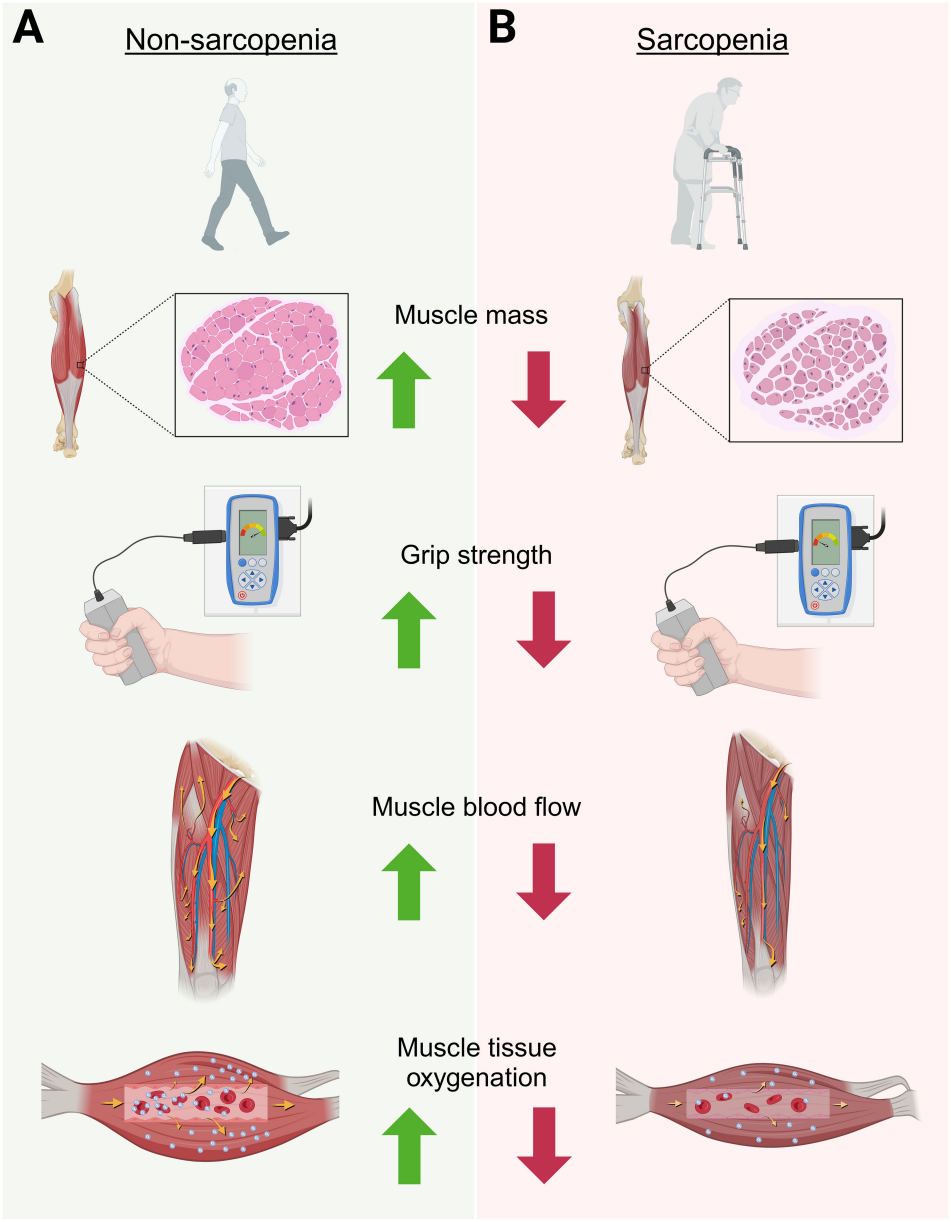
Graphical depiction highlighting the overall findings of this study on the physiological differences between non-sarcopenic **(A)** versus sarcopenic **(B)** muscle in older adults. This figure was Created in BioRender. Ruiz Caride, S. (2025).https://BioRender.com/h25h787.

The oxygenation capabilities and the maintenance of adequate blood flow is thought to be a key mechanism for muscle energy production and adequate delivery of nutrients to skeletal muscle (30). Additionally, the metabolic flexibility (i.e ability to adapt to available fuel sources) of the muscle may be in part linked to the vascular response to a stimulus. Therefore, understanding metabolic flexibility, muscle perfusion, and blood flow in aging adults may aid in the development of therapeutic recommendations to prevent progression of sarcopenia and frailty. The present study provides a novel approach to non-invasively examine differences in muscle perfusion and oxygenation between sarcopenic and non-sarcopenic older adults using NIRS. In our previous publication (15), we demonstrated distinct metabolic differences between sarcopenic and non-sarcopenic older adults, with sarcopenic individuals presenting with poor metabolic flexibility in the resting state as well as during exercise. We proposed that this metabolic inflexibility could be a mechanism underlying the losses of strength and physical function accompanying sarcopenia. As an extension to these findings, in our current study we identified additional differences in muscle perfusion and oxygenation between sarcopenic and non-sarcopenia older adults, especially during exercise. These distinct differences between sarcopenia and non-sarcopenia are displayed in **Figure 8**. While results from this pilot study does not indicate causality, it is reasonable to postulate that the previous finding of metabolic inflexibility, as indicated by impaired fat utilization, higher fasting glucose and respiratory quotient, and a blunted response to CHO utilization at rest and during exercise, together with reduced muscle perfusion and capillarization, may be key factors contributing to the progression of sarcopenia.

At rest, non-sarcopenic individuals experienced increases in ΔTHb_ATT_ following a meal stimulus (**Figure 3B**), with no change in deoxyHb (**Figure 3A**), which may be an indicator of increased muscle blood flow in response to meal induced insulin stimulus (5, 8). In contrast, sarcopenic individuals had no significant changes in ΔTHb_ATT_ (**Figure 3B**), indicating impaired skeletal muscle blood flow in response to an influx of nutrients, potentially indicating signs of reduced muscle perfusion at rest.

This impaired blood flow response in sarcopenic subjects was especially apparent during exercise. Sarcopenic individuals had consistently lower TSI during steady-state, aerobic exercise and showed no significant response in muscle blood flow during high intensity exercise compared to non-sarcopenic individuals, who demonstrated a decrease in muscle blood flow with intensity, as would be expected.

By design, the sarcopenic individuals in the present study demonstrated lower muscle mass, strength, and functionality than non-sarcopenic individuals, along with greater body fat percentage. This altered body composition most likely contributes to impaired glucose and insulin metabolism, which together with increased inflammation, can lead to endothelial dysfunction and hence blood flow. As demonstrated previously, this population of sarcopenic and non-sarcopenic older adults did demonstrate differences in glucose metabolism, insulin sensitivity, and metabolic flexibility (15). However, there were no differences in commonly assessed markers of inflammation such as CRP, TNF-α, and IL-6 between groups (unpublished), indicating that at least in this study population, inflammation does not seem to be a contributor. The increase in muscle blood flow observed in non-sarcopenic individuals may indicate that in those individuals with metabolic flexibility, the muscle is responsive to insulin stimuli, which may allow for greater muscle perfusion in response to nutrient delivery. This was not observed in sarcopenic individuals. Recently, Soares et al. (31) was able to use NIRS to detect differences in vascular responsiveness between lean and obese individuals during an oral glucose tolerance test (31). With the similarities between obesity/insulin resistance and the metabolic inflexibility that may be present with sarcopenia, these findings may indicate a reduced blood flow response post-prandial that may result in decreased nutrient delivery to the muscle cells which could lead to a decrease in energy availability and adaptations for muscular mass and strength.

Aging has been associated with mitochondrial dysfunction and impaired oxidative metabolism (32, 33). These impairments may be exacerbated with sarcopenia due to the important role skeletal muscle has on glucose metabolism and substrate utilization (34). Therefore, older adults with sarcopenia may experience metabolic impairments to a greater extent compared to non-sarcopenic older adults. It has been suggested that deoxyHb assessed by NIRS can detect differences in those with mitochondrial dysfunction compared to healthy individuals (35). This theory could potentially explain the decrease in deoxyHb post-prandial in sarcopenic individuals. Similarly, individuals with obesity demonstrated a decrease in muscle oxygen utilization (measured by deoxyHb via NIRS) 30- and 60-min post 75g of glucose consumption (7). With the potential for metabolic inflexibility of skeletal muscle developing within obesity and sarcopenia, this impairment in transitioning from fat to CHO oxidation could be reflected via NIRS measurements within the microvascular structure (7, 15).

During exercise, the oxygen and blood flow demands within the working skeletal muscle increase (36). Increased blood flow allows for greater Hb concentrations, thus leading to greater oxygen delivery to meet these enhanced requirements during exercise. With healthy aging, changes within the macro- and microvascular system occur, leading to metabolic impairments, as well as decreased exercise capacity (4, 37). With TSI as an indicator of oxygen delivery and utilization within the muscle, this study indicates a lower oxygen utilization during steady-state exercise in those with sarcopenia (**Figure 4**). Lower muscle tissue saturation has been demonstrated in older adults compared to young adults during a treadmill and a 6-min walking test, possibly tied to impaired microvascular function (38). Lower TSI in sarcopenic older adults, suggestive of reduced exercise adaptations, needs to be considered when developing exercise protocols for sarcopenic individuals. In addition, strategies to improve not only skeletal muscle mass, but also muscle blood flow should be a target for exercise interventions to help with this impaired oxygen delivery, which could help these individuals with improvements in physical capacity, reduced fatigue, and overall better quality of life (37, 39).

With the high prevalence of sarcopenia, determining nutrition and physical activity interventions to reduce the multitude of factors associated with sarcopenia is essential. Strength training is an effective strategy for improving skeletal muscle mass and strength; however, the anabolic response to exercise is weakened in older adults, potentially linked to this reduction in muscle perfusion. While increases in physical activity can improve this transport of nutrition and improve the anabolic response (30), this increased blood flow with exercise may not be as effective to those with reduced musculature. Moro et al. reported that older adults with lower skeletal muscle capillarization may have reduced muscular adaptations to strength training exercise (40). The present study indicated a similar, yet acute response to exercise, in which there was a lack of change in THb to fatiguing leg extension exercise in sarcopenic older adults compared to non-sarcopenic older adults (**Figure 5**). One potential theory is that having lower skeletal muscle consequently leads to lower capillarization, thus limiting the anabolic response from exercise that would result in skeletal muscle mass growth or maintenance to prevent or reduce progression of sarcopenia. Therefore, strategies to improve or prevent this decrease in capillarization may be a necessary part in utilizing nutrition and exercise to reduce the progression of sarcopenia, especially in those with severe sarcopenia.

While this exploratory study provides novel insight on NIRS responses between sarcopenic and non-sarcopenia at rest, post-prandial, and during exercise, this study had several limitations. While NIRS is a cost-effective, non-invasive tool that can be used in clinic, NIRS provides only an indirect measurement of blood flow and endothelial function compared flow mediated dilation using Doppler ultrasound. Although THb derived from NIRS has been reported to correlate with Doppler ultrasound blood flow (8), and there is a known association between NIRS-derived and flow-mediated dilation measurements of vascular responsiveness (41), utilizing results from NIRS alone should be interpreted with caution. NIRS also cannot reliably distinguish between Hb and Mb signals, which could affect accuracy of measurements of blood flow and tissue oxygenation. Additionally, while signals were normalized for adipose tissue, ATT still does affect signal quality, which is important to note provided the body composition differences among groups. On the NIRS device used in our study, the transmitting diodes and receiver are designed spatially to provide 3 source-detector distances of 30, 35, and 40 mm to allow for a depth sensitivity of up to 2.0 cm (5), which all participants were below, which helps account for this potential source of error, yet does still warrant caution in interpreting results. Additionally, because this was a pilot study with a small sample size, it was not possible to study impact of sex on the outcomes measured. Studies with larger sample sizes are warranted to address sex-related differences in metabolic outcomes tied to sarcopenia progression. Furthermore, since this study was a cross-sectional design, future research utilizing longitudinal or intervention designs is needed.

## 5 Conclusions

This exploratory study provides preliminary evidence that those with sarcopenia demonstrate decreased mitochondrial functionality, oxidative capacity, and blood flow that can be detected by non-invasive measurements using NIRS. While more research is needed in this area, the present study observed differences in NIRS responses between sarcopenic and non-sarcopenic individuals post-prandial and during exercise. Nutrition and exercise interventions focusing on strategies to improve blood flow during exercise, while promoting muscle protein synthesis are necessary to determine methods to reduce sarcopenia progression with aging.

## Data Availability

The raw data supporting the conclusions of this article will be made available by the authors, without undue reservation.
